# Supplementary material for: Multi‐dataset identification of innovative feature genes and molecular mechanisms in keratoconus
Source: J Cell Mol Med. 2024 Sep 19;28(18):e70079. doi: 10.1111/jcmm.70079 (PMC11412914; doi:10.1111/jcmm.70079)
Supplement: Supplementary file 2 — Material S2. [file JCMM-28-e70079-s002.zip › 7.GSEA analysis/4.MYRF/KEGG_GLYCOSAMINOGLYCAN_BIOSYNTHESIS_CHONDROITIN_SULFATE.html]

Details for gene set KEGG\_GLYCOSAMINOGLYCAN\_BIOSYNTHESIS\_CHONDROITIN\_SULFATE[GSEA]

|  || Dataset | MYRF.group.cls#h\_versus\_l |
| Phenotype | group.cls#h\_versus\_l |
| Upregulated in class | l |
| GeneSet | KEGG\_GLYCOSAMINOGLYCAN\_BIOSYNTHESIS\_CHONDROITIN\_SULFATE |
| Enrichment Score (ES) | -0.5751627 |
| Normalized Enrichment Score (NES) | -1.5186424 |
| Nominal p-value | 0.056338027 |
| FDR q-value | 0.19630218 |
| FWER p-Value | 0.739 |
Table: GSEA Results Summary

  

Fig 1: Enrichment plot: KEGG\_GLYCOSAMINOGLYCAN\_BIOSYNTHESIS\_CHONDROITIN\_SULFATE      
 Profile of the Running ES Score & Positions of GeneSet Members on the Rank Ordered List

  

| SYMBOL | TITLE | RANK IN GENE LIST | RANK METRIC SCORE | RUNNING ES | CORE ENRICHMENT || 1 | CHSY3 | na | 7748 | 0.144 | -0.1821 | No |
| 2 | B3GALT6 | na | 11357 | 0.073 | -0.2654 | No |
| 3 | XYLT1 | na | 14200 | 0.036 | -0.3372 | No |
| 4 | CHPF | na | 16697 | 0.011 | -0.4059 | No |
| 5 | B3GAT1 | na | 18510 | -0.005 | -0.4566 | No |
| 6 | CHSY1 | na | 19616 | -0.017 | -0.4835 | No |
| 7 | CHST13 | na | 20129 | -0.024 | -0.4916 | No |
| 8 | XYLT2 | na | 20667 | -0.030 | -0.4986 | No |
| 9 | B3GAT3 | na | 22420 | -0.051 | -0.5345 | No |
| 10 | B4GALT7 | na | 22839 | -0.057 | -0.5303 | No |
| 11 | DSE | na | 23802 | -0.072 | -0.5374 | No |
| 12 | CHST3 | na | 24034 | -0.076 | -0.5222 | No |
| 13 | CHST12 | na | 24199 | -0.079 | -0.5044 | No |
| 14 | B3GAT2 | na | 26655 | -0.127 | -0.5390 | Yes |
| 15 | CHST11 | na | 27685 | -0.152 | -0.5252 | Yes |
| 16 | CHPF2 | na | 27712 | -0.153 | -0.4823 | Yes |
| 17 | CHST15 | na | 28565 | -0.177 | -0.4564 | Yes |
| 18 | CHST7 | na | 30302 | -0.234 | -0.4397 | Yes |
| 19 | CSGALNACT2 | na | 31518 | -0.292 | -0.3916 | Yes |
| 20 | CHST14 | na | 33136 | -0.401 | -0.3240 | Yes |
| 21 | CSGALNACT1 | na | 34030 | -0.528 | -0.1991 | Yes |
| 22 | UST | na | 34622 | -0.769 | 0.0031 | Yes |
Table: GSEA details [plain text format]

  

Fig 2: KEGG\_GLYCOSAMINOGLYCAN\_BIOSYNTHESIS\_CHONDROITIN\_SULFATE      
 Blue-Pink O' Gram in the Space of the Analyzed GeneSet

  

Fig 3: KEGG\_GLYCOSAMINOGLYCAN\_BIOSYNTHESIS\_CHONDROITIN\_SULFATE: Random ES distribution      
 Gene set null distribution of ES for **KEGG\_GLYCOSAMINOGLYCAN\_BIOSYNTHESIS\_CHONDROITIN\_SULFATE**
